# Electroless Cobalt Deposition on Dealloyed Nanoporous Gold Substrate: A Versatile Technique to Control Morphological and Magnetic Properties

**DOI:** 10.3390/nano13030494

**Published:** 2023-01-26

**Authors:** Gabriele Barrera, Federico Scaglione, Federica Celegato, Marco Coïsson, Paola Tiberto, Paola Rizzi

**Affiliations:** 1 Istituto Nazionale di Ricerca Metrologica (INRIM), Str. delle Cacce, 91, 10135 Torino, Italy; 2 Dipartimento di Chimica e Centro Interdipartimentale NIS (Nanostructured Surfaces and Interfaces), Università di Torino, Via Pietro Giuria 7, 10125 Torino, Italy

**Keywords:** nanoporous gold, dealloying, electroless cobalt deposition, magnetic heterostructures, magnetic properties, first order reversal curves

## Abstract

The connection of multidisciplinary and versatile techniques capable of depositing and modeling thin films in multistep complex fabrication processes offers different perspectives and additional degrees of freedom in the realization of patterned magnetic materials whose peculiar physical properties meet the specific needs of several applications. In this work, a fast and cost-effective dealloying process is combined with a fast, low-cost, scalable electroless deposition technique to realize hybrid magnetic heterostructures. The gold nanoporous surface obtained by the dealloying of an Au_40_Si_20_Cu_28_Ag_7_Pd_5_ ribbon is used as a nanostructured substrate for the electrodeposition of cobalt. In the first steps of the deposition, the Co atoms fill the gold pores and arrange themselves into a patterned thin film with harder magnetic properties; then they continue their growth into an upper layer with softer magnetic properties. The structural characterization of the hybrid magnetic heterostructures is performed using an X-ray diffraction technique and energy-dispersive X-ray spectroscopy, while the morphology of the samples as a function of the electrodeposition time is characterized by images taken in top and cross-section view using scanning electron microscopy. Then, the structural and morphologic features are correlated with the room-temperature magnetic properties deduced from an alternating-gradient magnetometer’s measurements of the hysteresis loop and first order reversal curves.

## 1. Introduction

Structural and morphological alterations in magnetic thin films play a significant role in fine-tuning their magnetic properties, providing a wide range of opportunities to meet the specific needs of several studies and applications [1,2,3,4,5].

A multiplicity of advanced fabrication techniques have been designed, combined together, and successfully used to tailor magnetic thin films to usage needs. Such techniques are mainly based on top-down approaches such as lithography and etching or on bottom-up approaches involving deposition and self-assembly processes [5,6,7,8,9,10,11,12,13].

In view of a large-scale diffusion on the international market of devices based on patterned magnetic thin films, these fabrication techniques must also meet strict constraints related to versatility, environmental impact, and exceptional capabilities for low cost and mass production. In this framework, the electrodeposition technique and the dealloying process are effective in overcoming some of these constraints. The former is a fast, low-cost, scalable, nonvacuum electrochemical technique for growing metallic thin films with selectable thickness on any conductive substrate [14,15]. The latter is a common, fast and cost-effective process based on the selective etching from an alloy of the less noble elements followed by the self-assembly rearrangement by surface diffusion of the more noble metal into interconnected ligaments which lead to open pores with a random spatial arrangement [16,17,18,19]. These structural, compositional and morphological transformations induced by the dealloying process significantly affect the physical and chemical properties of the treated material [10,18,20,21]. The combined use of these two multidisciplinary and versatile techniques in multistep fabrication processes is expected to offer different perspectives and additional degrees of freedom in the realization of hybrid magnetic nanomaterials. 

In this work, the dealloying process of an Au_40_Si_20_Cu_28_Ag_7_Pd_5_ ribbon and the electrodeposition of the element Co are combined together to fabricate Co/Au magnetic heterostructures. In particular, the Au_40_Si_20_Cu_28_Ag_7_Pd_5_ ribbon, obtained using a rapid solidification technique, is subjected to the electrochemical dealloying process to selectively dissolve the less noble elements of the pristine alloy, obtaining a nanoporous gold (NPG) ribbon [22,23]. Subsequently, the NPG ribbon is used as the conductive substrate for the electrodeposition technique which allows the growth of a Co thin film on the nanostructured surface of the NPG pores, resulting in a hybrid Co/NPG magnetic heterostructure with unconventional shapes.

In recent decades, the electrodeposition of Co thin film on continuous Au electrodes has been extensively investigated for its wide-range synergistic or complementary physical and chemical properties which constitute the core of interesting scientific studies and applications [24,25,26,27,28].

Initially, several studies were conducted to understand the nucleation and growth mechanisms of Co on continuous Au surfaces in different solutions using potential step techniques and cyclic voltammetry [27,29,30], paying particular attention to the resulting magnetic properties [31].

Recently, advanced studies have been performed on complex Co/Au multilayer structures investigating their hybrid physical properties such as the spin reorientation transition effect [32], magneto-optic surface plasmon resonance behavior [33,34] and high-order ferromagnetic resonances [28]. Furthermore, in more application-oriented studies, Co-oxide layers deposited on nanoporous gold substrates have shown highly effective activities for the electrocatalytic reduction of peroxide [35] and high sensing performance for nonenzymatic glucose [36].

In the present study, hybrid Co/NPG magnetic heterostructures were fabricated with different thicknesses of Co layer on the NPG substrate by properly tuning the electrodeposition time. The porous structure of the gold substrate leads to a two-step growth of the Co magnetic layer: first, the Co atoms fill the gold pores forming a patterned magnetic Co layer constituted by separated magnetic islands; then, after the pores have been plugged, the electrodeposition continues in a continuous Co layer. The structural characterization of the hybrid magnetic heterostructures is performed using an X-ray diffraction (XRD) technique and energy-dispersive X-ray spectroscopy (EDS). The evolution of the morphological properties of the Co/NPG magnetic heterostructures as a function of the electrodeposition time is properly characterized using scanning electron microscopy (SEM). In addition, the room-temperature magnetic properties are investigated using a high-sensitive alternating gradient field magnetometer (AGFM) to measure hysteresis loop and first order reversal curves (FORC) to achieve an in-depth description of the magnetization reversal processes in the hybrid Co/NPG magnetic heterostructures.

This work aims to contribute to the current technical challenge of combining rapid and inexpensive, scalable, nonvacuum techniques to obtain hybrid magnetic nanostructured materials with tunable magnetic properties from high saturation and low coercivity to low saturation and high coercivity.

## 2. Experimental

### 2.1. Synthesis of NPG Substrate 

An ingot of the master alloy of composition Au_40_Si_20_Cu_28_Ag_7_Pd_5_ (at.%) was prepared by arc-melting pure elements in a Ti-gettered Ar atmosphere. Then an amorphous ribbon 2 mm wide and 25 μm thick was obtained by spinning the molten alloy onto a copper wheel rotating at a speed of 25 m/s. Samples of 30 mm in length were cut from the ribbon. EDS analysis of the as-spun ribbon is in good agreement with the nominal composition and is reported in Figure S1 of the Supplementary Materials.

Nanoporous gold (NPG) was obtained by electrochemical dealloying of the ribbons in a three-electrode cell (Pt counter-electrode, saturated Ag/AgCl double-bridge reference electrode, and the sample as working electrode). The electrolyte and etching conditions, i.e., 1.05 V at the temperature of 70 °C in 1 M sulphuric acid (Merck, Darmstadt, Germany), were selected from a previous work [22]; after 6 h of treatment the sample achieves a complete dealloying through the whole thickness of the ribbon.

### 2.2. Electroless Co Deposition on NPG

The electrolyte used for the electroless Co deposition was freshly prepared before the deposition and composed of 0.177 M KNaC_4_H_4_O_6_·4H_2_O, 0.084 M CoCl_2_·6H_2_O and 0.25 M NaOH (Merck, Darmstadt, Germany) [37]. Compounds were mixed one by one in ultra-pure distilled water under stirring condition; the solution is ready to be used when all salts have been dissolved and the color turns to violet. 

The NPG was cleaned in distilled water before the experiment and then contacted with a cylindrical specimen of pure aluminum (9 mm diameter and 60 mm length); then, both electrodes were immersed in the solution. The electroless Co plating process is induced by the dissolution of the Al rod that works as an anode: $\mathrm{Al}+4{\mathrm{OH}}^{-}\to {\left[\mathrm{Al}{\left(\mathrm{OH}\right)}_{4}\right]}^{-}+3e^{-}$. Electrons generated by the oxidation of the Al rod flow through the connection wire to the NPG discharging the Co ions in the solution as metallic cobalt inside the pores and onto the NPG working as a cathode: ${\mathrm{Co}}^{2+}+2e^{-}\to \mathrm{Co}$.

The electroless deposition process is a plating technique in which crystals of pure Co nucleate and grow on the available surface area of the conductive NPG substrate by coating it. The Co layer is attached to the Au surface, but the two elements are distinct and not combined in a solid solution. Using this method, the hybrid Co/NPG magnetic heterostructures are obtained.

The lengths of the immersed NPG and Al rod and the amount of electrolyte were kept constant to guarantee the reproducibility of the experiments. The electroless deposition time (t_dep_) was selectively tuned in the range 1–195 min. The studied samples were called Co-NPG_t_dep_. A picture of the experimental setup and a scheme of the experiment are shown in Figure S3 in the Supplementary Materials.

All samples were deeply rinsed with ultrapure water to remove the excess of acid solution inside the pores and then air-dried before further experiments.

### 2.3. Morphological, Structural and Magnetic Characterization of the Hybrid Co/NPG Heterostructures 

The morphology and stoichiometry of the hybrid Co/NPG heterostructures were studied using scanning electron microscopy (SEM) with both secondary electron (SE) and back scattered electron (BSE) detectors (Inspect SEM, FEI) (FEI, Hillsboro, OR, USA). Moreover, the SEM is also equipped with an EDS detector used for the elemental analysis of the sample composition. Cross-sections of NPG and Co/NPGs were obtained by gripping each sample at both extremities with flat-tip tweezers and bending them until they fractured. 

The dimensions of ligaments of the NPG were measured at their narrower necks using Leica software (version 4.2.0, 2003–2012, Leica Microsystem, Wetzlar, Germany) [38].

The structural characterization of the hybrid magnetic heterostructures was performed using an X-ray diffraction technique (XRD) by using a Panalytical X-pert X-ray Diffractometer in Bragg–Brentano geometry (Panalytical, Almelo, The Netherlands) and equipped with a monochromatic Cu Kα radiation. The set-up for diffraction experiments was 30 kV, 40 mA in the region 35–85 degrees in 2ϑ. Compositional analysis was conducted using energy-dispersive X-ray spectroscopy (EDS) equipment with an EDS probe (Oxford Ultim-Max 100) (Oxford Instruments, Abingdon, UK).

The room-temperature magnetic properties of the Co/NPG heterostructures were investigated using a high-sensitive alternating gradient field magnetometer (AGFM) (Princeton measurements corporation) (Princeton, NJ, USA). Hysteresis loops were measured by applying a magnetic field in the range ± 10 kOe. Additionally, first order reversal curves (FORC) were performed to achieve a more detailed description of the magnetization process. In detail, FORC diagrams allow us to bring out the coexistence and interaction of different magnetization reversal modes, to distinguish between the reversible and irreversible magnetization mechanisms, and to evaluate the distribution of coercivity. FORC measurements are obtained according to the following routine: (i) the sample is completely saturated by a proper value of applied field (H_a_); (ii) H_a_ is decreased to a reversal field H_R_; and (iii) the magnetization (M) of the sample is measured while increasing H_a_ from H_R_ back to the saturating value. The sequence is repeated for many different values of H_R_.

Subsequently, the FORC distribution is calculated as the mixed second derivative of the magnetization with respect to the applied field H_a_ and the reversal field H_R_ [39]:

(1) $$\rho \left(H_{a},H_{R}\right)=-\frac{1}{2}\frac{\partial^{2}M\left(H_{a},H_{R}\right)}{\partial H_{a}\partial H_{R}}$$

When plotting a FORC distribution, a coordinate change is usually made from (H_a_, H_R_) to (H_B_ = (H_a_ + H_R_)/2, H_C_ = (H_a_ − H_R_)/2), where H_B_ is called the bias field, and H_C_ is the coercive field. Nonzero values of ρ indicate (H_a_, H_R_) values where irreversible magnetization processes take place.

## 3. Results and Discussion

**Figure 1:**
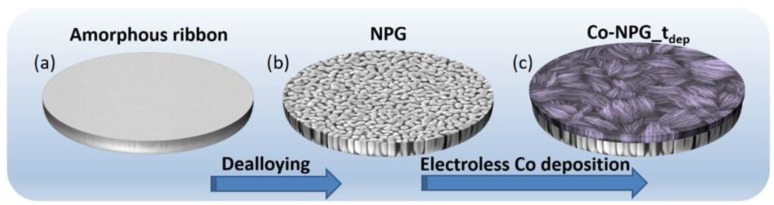
Scheme of the procedure followed to obtain Co-NPG_t_dep_. (**a**) Synthesis of the amorphous ribbon through melt spinning process; (**b**) dealloying process to obtain NPG; (**c**) electroless Co deposition onto NPG at selected times.

The preparation of Co-NPG_t_dep_ samples is realized in a three-step procedure, the scheme of which is described in Figure 1. First, (a) the amorphous ribbon is obtained using a melt spinning process of the master alloy ingot, then (b) it is subjected to the dealloying process to obtain a fully dealloyed NPG, and finally, (c) the electroless deposition of Co onto NPG is performed to different extents by selecting the deposition time. 

**Figure 2:**
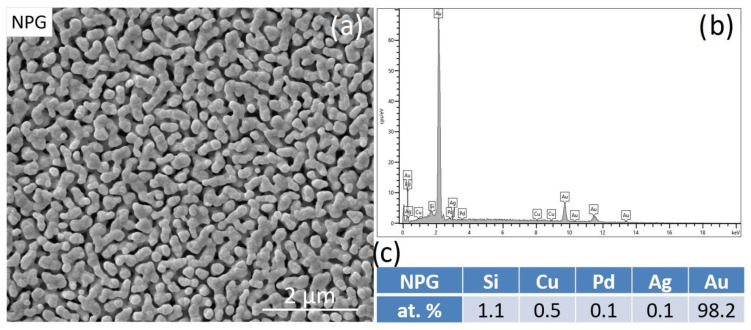
(**a**) SE-SEM image, (**b**) EDS spectrum and (**c**) composition in at. % of the NPG obtained by dealloying of the amorphous precursor.

The morphology of the freshly prepared dealloyed NPG, analyzed using secondary electrons SE-SEM, is characterized by islands of pure Au with sub-micrometric size and irregular shape [22]. A well-defined porous structure develops among the micro-islands. EDS analysis of NPG reports that the ligaments are composed of almost pure Au with a very small amount of retained atoms (Figure 2b,c). 

### 3.1. Morphology and Structural Characterization of Co/NPG Heterostructures

**Figure 3:**
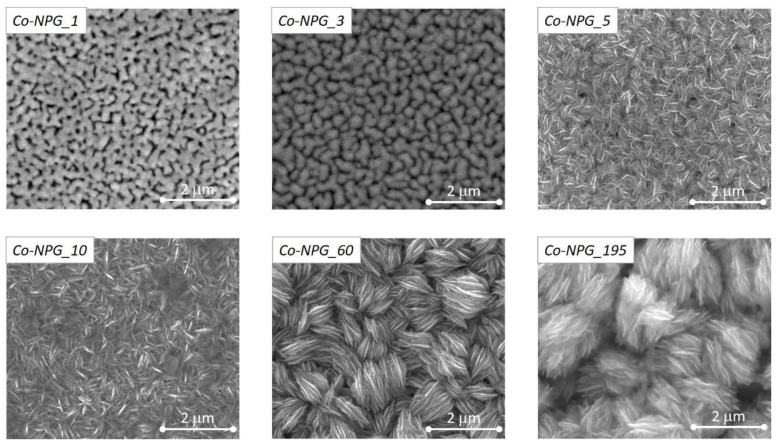
SE-SEM top-view image of the morphology of all studied hybrid Co/NPG heterostructures.

The evolution of the morphology of the Co/NPG heterostructures as a function of deposition time was investigated using SE-SEM; the micrographs of the top view surface are shown in Figure 3.

Clearly, the sample morphology strongly depends on t_dep_, the increase of which initially induces a progressive coverage and plugging of the ligaments and pores of the pristine NPG substrate, then leads to the deposition of an upper continuous Co thin film characterized by fine lamellar deposits of cobalt organized in microseed structures.

In particular, the top-view morphology of the Co-NPG_1 sample shows a homogeneous ligament–pore structure similar to that of the underlying NPG substrate (see Figure 2a), indicating that the deposition of the Co atoms mimics the NPG morphology by penetrating inside the gold pores and covering the pristine ligaments. Similarly, the morphology of the Co-NPG_3 sample is still characterized by ligament–pore structure, although the edges of the ligaments appear more rounded and less defined, whereas the pores are smaller in size.

This evidence reveals how the Co electrodeposition begins (t_dep_ ≤ 3 min) by mimicking the porous Au structure with a gradual coverage of ligaments and progressive obstruction of pores. Therefore, as a function of the deposition time, the formation of a nanopatterned Co layer composed of micro-islands interpenetrated in the NPG substrate occurs.

The complete plugging and coverage of the NPG substrate by Co atoms are observed in the Co-NPG_5 sample and in all the others obtained with an even longer deposition time. In particular, these samples show a progressive formation of a lamellar Co structure organized in interconnected bundles randomly oriented in the layer plane. The lamellae and bundles increase in size as a function of t_dep_ and as a result are well-defined in the Co-NPG_60 and Co-NPG_195 samples.

**Figure 4:**
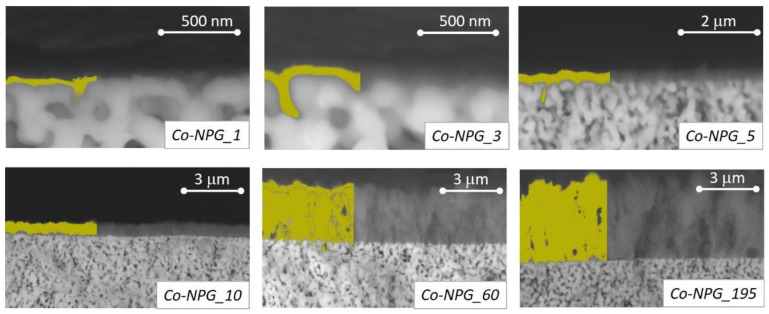
BSE-SEM cross-section images of the morphology of all studied hybrid Co/NPG heterostructures. The images are taken using backscattered electrons. The yellow color is used to emphasize the Co layer.

Figure 4 shows a cross-section view of all the samples. In this case, the images were taken using high-energy backscattered electrons (BSE) in order to highlight, with the gray scale, the distribution of the multiple elements that make up the sample. Taking advantage of this, in the left portion of each image, a contiguous region based on the same shade of gray was automatically selected and colored in yellow by software to emphasize the electrodeposited cobalt layer (darker gray) on the NPG substrate (clearer gray).

In the Co-NPG_1 and Co-NPG_3 samples, the penetration of the Co atoms in the pores is limited to about one hundred nanometers below the upper surface of the NPG substrate, leading to the formation of the micro-islands that compose the nanopatterned Co layer inside the NPG substrate. The thickness of the Co layer, estimated from the cross-section SEM images of Figure 4, is roughly 0.03 and 0.06 μm for Co-NPG_1, Co- and Co-NPG_3 samples, respectively. In all other samples obtained with longer deposition times, the cross-section images clearly confirm that the Co deposition completely blocked the pores of the NPG substrate and continued forming on its surface a continuous Co layer whose thickness increases as the deposition time is increased. In particular, the thickness of the continuous Co layer is roughly estimated at 0.32, 0.75, 3.0 and 4.7 μm for Co-NPG_5, Co-NPG_10, Co-NPG_60 and Co-NPG_195 samples, respectively.

**Figure 5:**
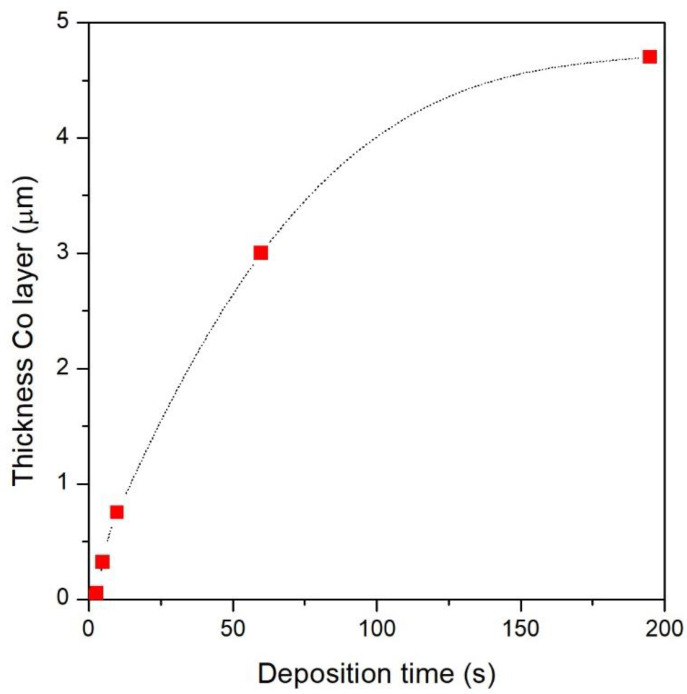
Evolution of the thickness of the Co layer as a function of deposition time.

The thickness of the Co layer as a function of the deposition time is shown in Figure 5. As highlighted, the deposition rate is not constant but slows down progressively. This evidence could be related to the progressive reduction of the conductive surface of the working electrode, which changes from the large surface of the NPG to a smaller one during the growth of the Co layer.

In brief, the Co layer interpenetrates the porous structure of the NPG substrate in the first stage of the electrodeposition (t_dep_ ≤ 3 min), leading to a magnetic nanostructured layer. For t_dep_ > 3 min, the deposition continues in a continuous Co layer on top of the nanostructured one, forming a hybrid magnetic bi-layer structure.

**Figure 6:**
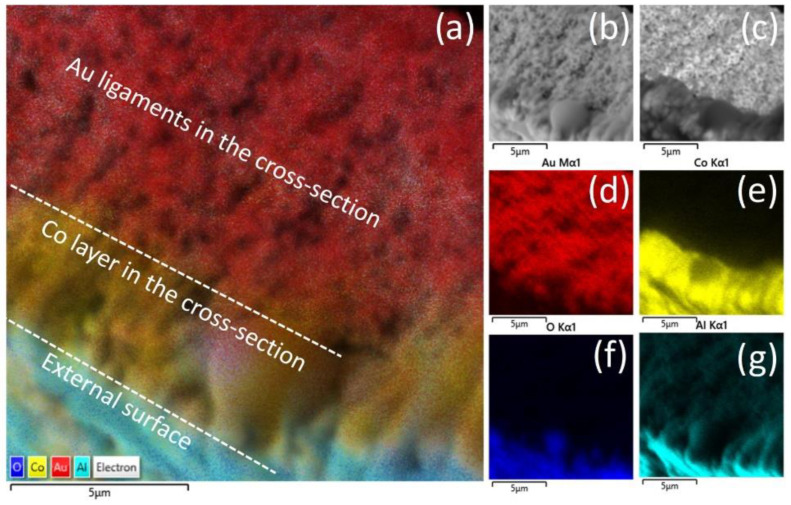
(**a**) Layered compositional map depicting Au, Co, O and Al content of the sample Co-NPG_195; (**b**) SE-SEM image, (**c**) BS-SEM image; (**d**) map showing only Au content; (**e**) map showing only Co content; (**f**) map showing only O content; and (**g**) map showing only Al content.

The EDS spectrum of Co-NPG samples was evaluated on the top view surface and an elemental map distribution is provided for the cross-section. Co-NPG_195 was selected as representative of all electroless Co deposited samples. The EDS spectrum in Figure S2 of the Supplementary Material reports the presence of Cl and Al as residual contaminations of the electroless deposition; the signal of carbon may be due to leftover KNaC_4_H_4_O_6_·4H_2_O used for the preparation of the electrolyte. Instead, the peak of oxygen suggests the formation of a thin native oxide layer that forms after deposition on top of the metallic cobalt layer. Evidence for this is corroborated by the elemental map of the cross-section in Figure 6: while the Al and O atoms are mainly accumulated in the external surface (in blue and cyan respectively in Figure 6f,g), the inner deposited Co (in yellow in Figure 6e) is not combined with oxygen.

**Figure 7:**
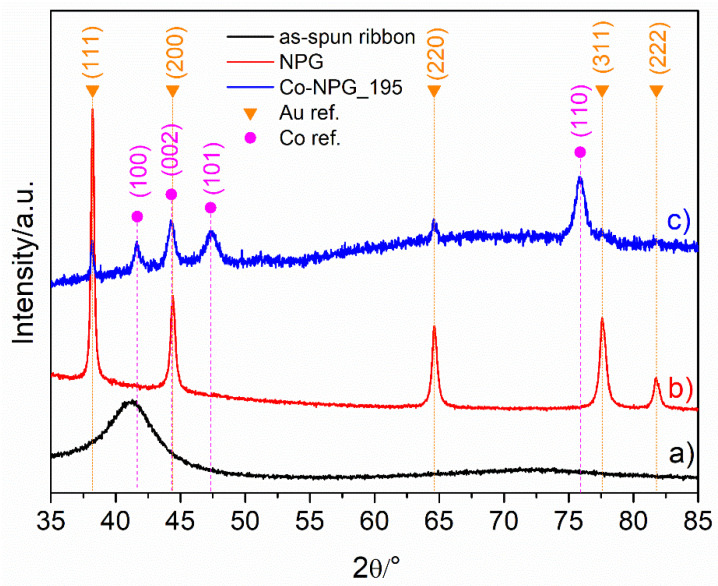
XRD patterns given by the as-spun ribbon (**a**), NPG (**b**) and Co-NPG_195 (**c**).

Finally, the structural characterization of the as-spun ribbon, NPG, and Co-NPG was conducted using X-ray diffraction and reported in Figure 7. The as-spun ribbon shows the typical halo of an amorphous structure; after dealloying, the amorphous structure is disrupted and the NPG pattern displays the diffraction peaks of an almost pure Au fcc phase. In the Co-NPG_195 pattern, the reflections can be indexed to crystalline cobalt with hcp phase, in some cases super-imposed on the Au fcc reflections of NPG underneath; reflections associated with cobalt oxides are not present, i.e., the native oxide layer on the sample surface (see Figure 6) is not detectable.

### 3.2. Magnetic Properties of Co/NPG Heterostructures 

**Figure 8:**
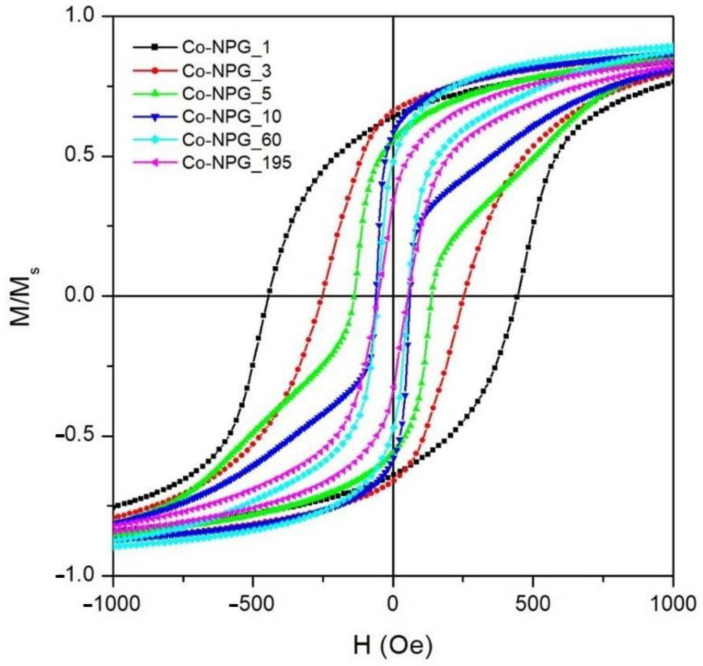
Room-temperature hysteresis loops of all the studied hybrid Co/NPG heterostructures.

The room-temperature hysteresis loops of the Co/NPG heterostructures are shown in Figure 8. The magnetic field is applied in the sample plane and the M(H) curves are normalized to the magnetization value at H = 10 kOe to make the comparison easier. Each studied sample exhibits a well-defined hysteretic curve that reaches full magnetic saturation at a high field (H = 10 kOe) (see Figure S4 in Supplementary Material). This evidence is fully compatible with the deposition of the ferromagnetic Co layer. Consequently, the presence of Co-oxide impurities, typically characterized by paramagnetic nonsaturating behavior at room temperature [40,41,42], is not detected; this result is compatible with the mere presence of the thin native Co-oxide on top of the hybrid-heterostructures. This magnetic evidence is in perfect agreement with the results of the EDS and XRD characterizations (see previous Section 3.1).

The reversal process of magnetization in the Co/NPG heterostructures was strongly affected by deposition time. The hysteresis loop of the Co-NPG_1 sample displays a single reversal mechanism of magnetization which gradually moves from positive to negative saturation (and vice versa); the normalized remanence (M_r_/M_s_) and the coercive field (H_c_) are 0.64 and 445 Oe, respectively.

These magnetic features of the Co-NPG_1 sample arise from the nanostructured morphology of the Co layer electrodeposited inside the NPG substrate (see Figure 3 and Figure 4). In fact, the pattern of micro-islands induces local magnetic anisotropies that compete with the crystal ones hindering the domain wall motion and the rearrangement of the magnetic domains, resulting in a harder magnetic behavior with respect to the other samples. 

Conversely, the Co-NPG_195 sample, i.e., the one with the thickest continuous Co layer in the hybrid magnetic structure, is characterized by a reversal of magnetization that occurs in a magnetic field range of about ±1 kOe; a slow reduction of the magnetic moment from magnetic saturation to a remanence state (M_r_/M_s_ ≈ 0.33) is followed by a prompt inversion of the magnetization in a single jump around the coercive field (H_c_ ≈ 52 Oe), indicating a rearrangement of the magnetic domain in the upper continuous Co layer. The reduction of M_r_/M_s_ and H_c_ values compared to those of the Co-NPG_1 sample indicates that, in the thicker sample, the magnetization reversal process is mainly dominated by the rearrangement of the magnetic domains in the upper continuous Co layer which acts a lower effective magnetic anisotropy, whereas the magnetic contribution of the underlying nanopatterned Co layer is almost negligible.

Interestingly, the Co-NPG_5 and Co-NPG_10 samples reveal completely different magnetic behavior. Both samples display a two-mechanism magnetization reversal, proving that the inversion processes of magnetization in the nanopatterned and continuous layers occur at different field values and with almost equal intensity. In particular, the first mechanism, present at a low magnetic field, is a clear switching step of magnetization related to the domain rearrangement in the upper continuous softer magnetic layer; conversely, the second one, characterized by a slow approach of the magnetization toward saturation, is due to the magnetization orientation in the micro-islands of the nanopatterned harder magnetic layer. 

**Figure 9:**
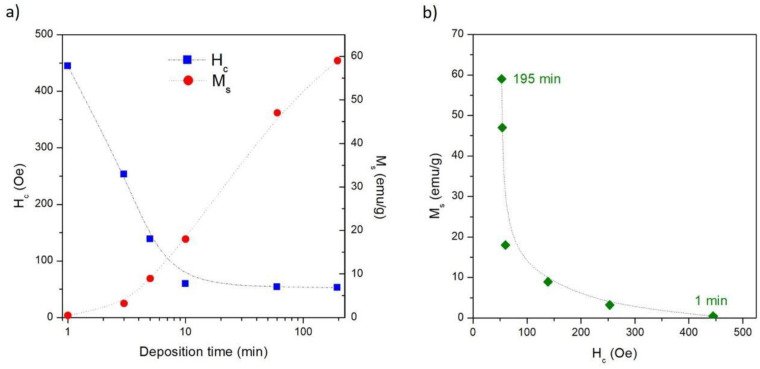
(**a**) Evolution of coercive field (H_c_) and saturation magnetization (M_s_) as a function of electrodeposition time; (**b**) saturation magnetization (M_s_) as a function of coercive field (H_c_) for all studied hybrid Co/NPG heterostructures. The dashed lines are only a guide for eyes.

The evolution of the coercive field as a function of the deposition time is shown in Figure 9a (blue squares). As already mentioned, the hindering of the magnetization reversal induced by the micro-islands in the nanopatterned Co-NPG_1 sample results in the highest value of coercive field. Increasing t_dep_ up to 10 min, H_c_ values are observed to swiftly decrease, confirming the gradual softening of the overall effective anisotropy in the samples induced by the progressive growth of the upper continuous Co layer. For electrodeposition times longer than 10 min, the coercive field remains almost constant, indicating that the magnetic reversal processes in these samples are mainly governed by the upper Co continuous layer independently of its thickness.

The evolution of the saturation magnetization (*M_s_*) as a function of the deposition time is also shown in Figure 6a (red bullets). The *M_s_* values are obtained by dividing the measured magnetic signal by the overall mass of the heterostructure, which includes both the NPG substrate and the electrodeposited Co layer. As expected, the *M_s_* value monotonically increases as a function of deposition time because the amount of Co progressively increases with respect to the constant amount of NPG. Ideally, the *M_s_* vs. t_dep_ curve asymptotically tends to the saturation value of bulk Co. However, the nonlinear evolution of *M_s_* as a function of t_dep_ confirms that the Co deposition rate is not constant (see Figure 5).

In Figure 9b, the *M_s_* value is reported as a function of *H_c_*, both obtained from the hysteresis loop for each Co-NPG sample. These two magnetic quantities draw the fingerprint on which the magnetic properties of the hybrid Co/NPG heterostructures can be tuned by the deposition time. This result is due to the strong correlation between the magnetic properties and the evolution of the morphologic structure in the magnetic heterostructures. In summary, the electrodeposition of Co on a NPG substrate is able to provide samples with magnetic properties ranging from high saturation and low coercivity to low saturation and high coercivity.

Such effects on the magnetic properties induced by the deposition of Co on the NPG substrate are in agreement with results obtained in similar systems using different patterned substrates [43,44,45,46]. Diblock-copolymer porous templates arranged in anti-dots arrays [43], copper substrates patterned by mechanical scratches [46], spin-coated colloidal templates [45] and wrinkled polydimethylsiloxane (PDMS) elastomeric templates [44] are some published examples of studies using patterned substrates to tune the effective magnetic anisotropy in the Co layer, resulting in variation of its magnetic properties.

In all these systems, the magnetic anisotropy variation correlated with morphological modification in the Co layer induced by the underlying patterned substrate leads to an increase in the value of the coercive field compared with one of the same Co layer deposited on a flat substrate. Similar results are observed in the samples studied in this work; H_c_ increases monotonically from the sample Co-NPG_1 (the sample most influenced by the NPG substrate) towards the sample Co-NPG_195 (least affected by the NPG substrate) (Figure 6a). Satisfactory agreement is also observed for the value of M_r_/M_s_ [46].

**Figure 10:**
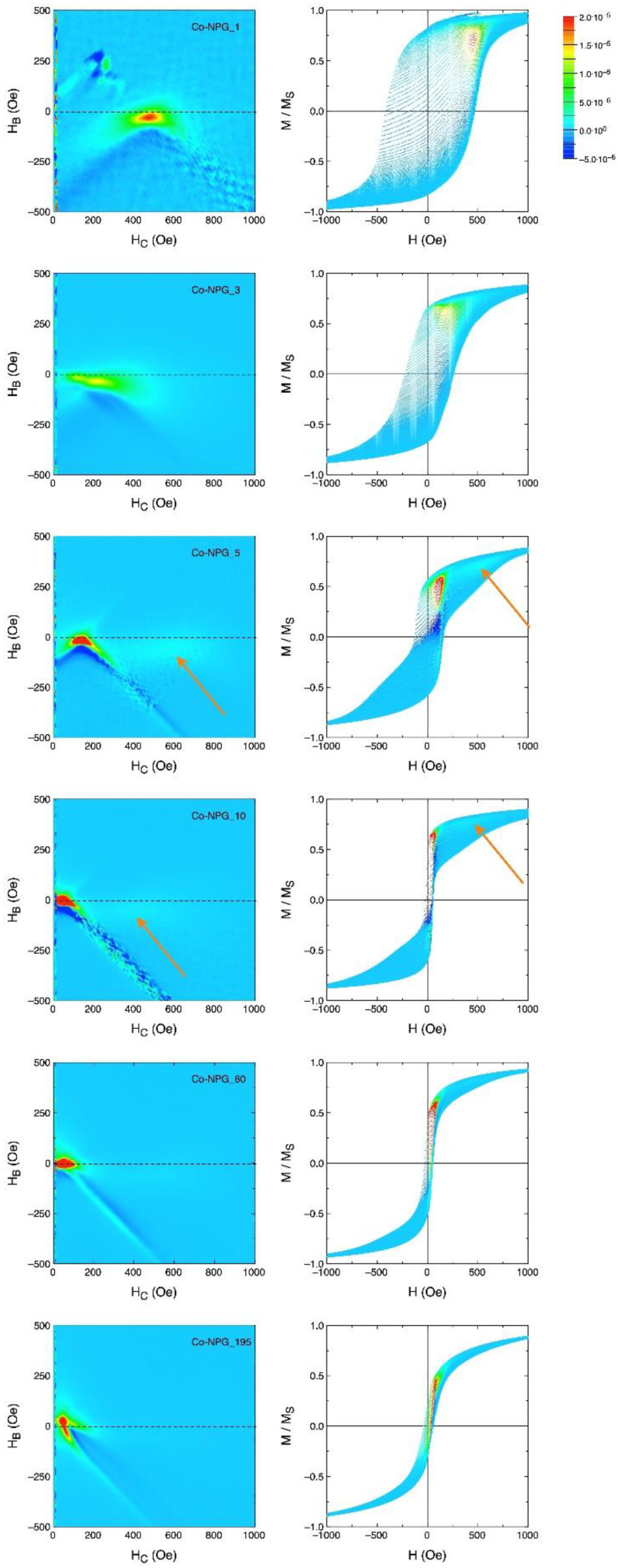
Left panels: FORC distribution in the (H_B_,H_C_) plane for all studied Co-NPG_X samples; right panels: subset of the measured reversal curves.

A more detailed description of the magnetization process is provided by the measurement of the first order reversal curves (FORC) [47,48,49,50]. The resulting reversal distribution is plotted as a function of the coercive field H_c_ and of the bias (interaction) field H_B_ according to the normalized color scale (arbitrary units) shown in Figure 10. In the same figure, beside the FORC diagram, the reversal curves are also plotted with individual measurement points represented in the same color scale, to show the regions of the loop areas that are responsible for the most important irreversible reversal processes.

The Co-NPG_1 sample is characterized by a sharp peak at the coercive field. The green halo extending to its left and right indicates that a relatively narrow distribution of coercive fields can be found in the sample. This suggests that the micro-islands obtained by electrodeposition into nanoporous gold are rather homogeneous in magnetic anisotropy, and behave collectively under the application of the external magnetic field. A small degree of interaction among them is marked by the shift of the FORC peak at slightly negative values of H_B_, together with the faint blue (negative) region at the bottom of the FORC peak, representative of weak, local exchange interactions [39,51,52,53].

The Co-NPG_3 is instead characterized by a much wider distribution of coercive fields: in fact, the FORC distribution has a very broad peak not centered at the exact value of the coercive field of the loop, indicating that the micro-islands in the sample are now less homogeneous, and present a distribution of sizes and anisotropies that contribute, all with approximately the same weight, to the overall shape of the hysteresis loop. As for the Co-NPG_1 sample, the FORC distribution is again slightly shifted at negative values of H_B_, indicating weak magnetic exchange interactions among the Co micro-islands.

The Co-NPG_5 sample is instead characterized by a sharp peak at a much lower coercive field than that of the Co-NPG_1 sample, due to the Co continuous layer on top of the NPG substrate. However, a very faint, broad peak (the region of almost white color indicated by the orange arrow in Figure 10) is also present at much higher coercive field values. This characteristic clearly marks the presence of two different and uncoupled magnetic entities in this sample: the Co micro-islands in the nanoporous NPG substrate are still responsible for the high-field FORC peak, which also appears at an H_c_ value comparable to that of the sample Co-NPG_1, confirming that the two magnetization reversal processes appearing in the Co-NPG_5 sample are uncoupled. Additionally, these peaks keep on displaying a slight bias at negative H_B_ values. The continuous Co layer at the top now has a thickness that is sufficient to drive the majority of the reversal processes of the sample: in fact, the coercive field of the loop corresponds to the much stronger peak at approximately 200 Oe. The blue (negative) region just below the main peak indicates that the magnetic material responsible for this portion of the hysteresis loop is dominated by strong exchange interactions, as expected for a ferromagnetic film.

The Co-NPG_10 sample is qualitatively similar to the Co-NPG_5 one, with a still more increased role of the low-field reversal contribution of the continuous Co layer with respect to Co micro-islands, whose faint and broad peak in the FORC distribution is now barely visible (almost white region indicated by the orange arrows in Figure 10). This faint coercivity peak ascribed to the Co micro-islands appears at lower values compared with the samples with shorter deposition times, indicating that the continuous Co layer at the top is now able to partially drive the reversal of the magnetization of the Co micro-islands underneath.

The Co-NPG_60 sample looks almost identical to the Co-NPG_10 one except that the faint halo in the FORC distribution at high fields is now practically invisible, overcome by the much stronger contribution of the continuous Co film; this makes the Co micro-islands’ contribution to the total magnetization curve negligible, and their magnetization reversal probably almost completely driven by the interaction with the continuous Co top layer.

Finally, the Co-NPG_195 sample is characterized by a much narrower loop, with a very sharp peak close to the origin of the axes, indicating a very narrow distribution of coercive fields, and a magnetization reversal process that involves the whole volume of the magnetic material at the same time.

## 4. Conclusions

Multidisciplinary and versatile techniques such as the electrochemical dealloying process and electrochemical deposition are successfully combined to fabricate hybrid magnetic heterostructures. The dealloying process selectively dissolves the less noble elements from the Au_40_Si_20_Cu_28_Ag_7_Pd_5_ ribbon, producing a nanoporous gold (NPG) ribbon which is subsequently used as the conductive substrate for the electroless Co deposition.

The electrodeposition time proved to be a free parameter suitable for fine-tuning and controlling the morphological features and the magnetic properties of the hybrid Co/NPG heterostructures, which appear to be closely related to each other.

A patterned magnetic Co layer consisting of magnetic islands penetrated within the ligaments and pores of the pristine NPG substrate is obtained using short deposition times (t_dep_ ≤ 3 min); the corresponding magnetic properties are dominated by local magnetic anisotropies inside the patterned structure, leading to a single magnetization reversal mechanism with the highest coercive and normalized remanence values compared with the other samples. 

After the plugging of the NPG-substrate pores by cobalt (t_dep_ ≥ 5 min), the morphology of the Co/NPG heterostructure progresses into a continuous Co layer consisting of a lamellar structure organized in interconnected and randomly oriented bundles which are responsible for softer magnetic properties. Consequently, the presence of two different and uncoupled magnetic behaviors is clearly observed until the softer magnetic signal arising from the continuous upper Co layer becomes predominant.

Therefore, the peculiar magnetic properties of the studied hybrid Co/NPG heterostructures, ranging from high saturation and low coercivity to low saturation and high coercivity, originate from the synergistic combination of the NPG substrate morphology and conformal Co electrodeposition.

## Data Availability

All data are available from the authors. Please contact the corresponding authors.

## Supplementary Materials

The following are available online at https://www.mdpi.com/article/10.3390/nano13030494/s1, Figure S1: EDS spectrum and table with the composition in at.% of the as-spun ribbon. Figure S2: EDS spectrum of the Co-NPG_195 sample. Figure S3: Picture of experimental setup for electroless Co deposition on NPG substrate; Figure S4: large-scale plot of hysteresis loop at room temperature for all studied hybrid samples.
