# Supplementary material for: Electroless Cobalt Deposition on Dealloyed Nanoporous Gold Substrate: A Versatile Technique to Control Morphological and Magnetic Properties
Source: Nanomaterials (Basel). 2023 Jan 26;13(3):494. doi: 10.3390/nano13030494 (PMC9920968; doi:10.3390/nano13030494)
Supplement: Supplementary file 1 [file nanomaterials-13-00494-s001.zip › nanomaterials-2099835-Suplementary.pdf]

Supplementary Materials

Electroless Cobalt deposition on dealloyed nanoporous Gold substrate: a versatile technique to control morphological and magnetic properties

Gabriele Barrera[1]*, Federico Scaglione[2]*, Federica Celegato[1], Marco Coïsson[1], Paola Tiberto[1] and Paola Rizzi[2]

[1]Istituto Nazionale di Ricerca Metrologica (INRIM), Str. delle Cacce, 91, 10135 Torino, Italy
[2]Dipartimento di Chimica e Centro Interdipartimentale NIS (Nanostructured Surfaces and Interfaces), Università di Torino, Via Pietro Giuria 7, 10125 Torino, Italy

* Correspondence: g.barrera@inrim.it; federico.scaglione@unito.it.

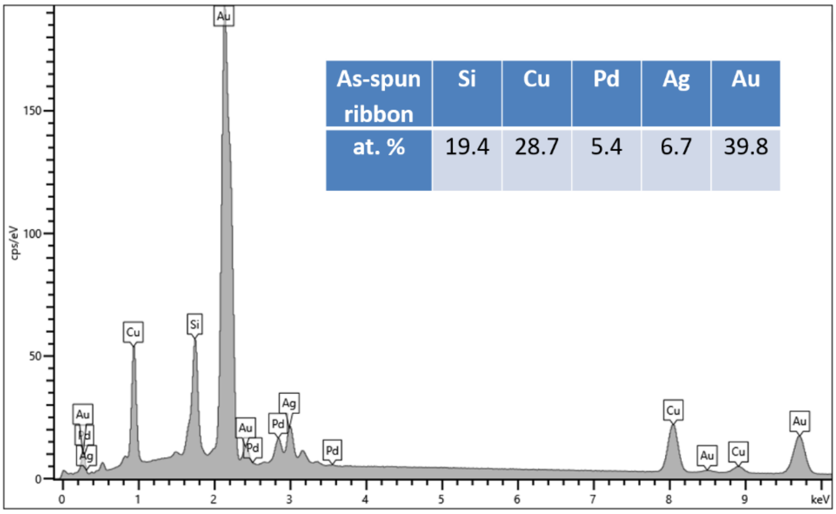

As-spun ribbon	Si	Cu	Pd	Ag	Au
at. %	19.4	28.7	5.4	6.7	39.8

Figure S1. EDS spectrum and table with the composition in at.% of the as-spun ribbon. Compositional result is in good agreement with the nominal one.

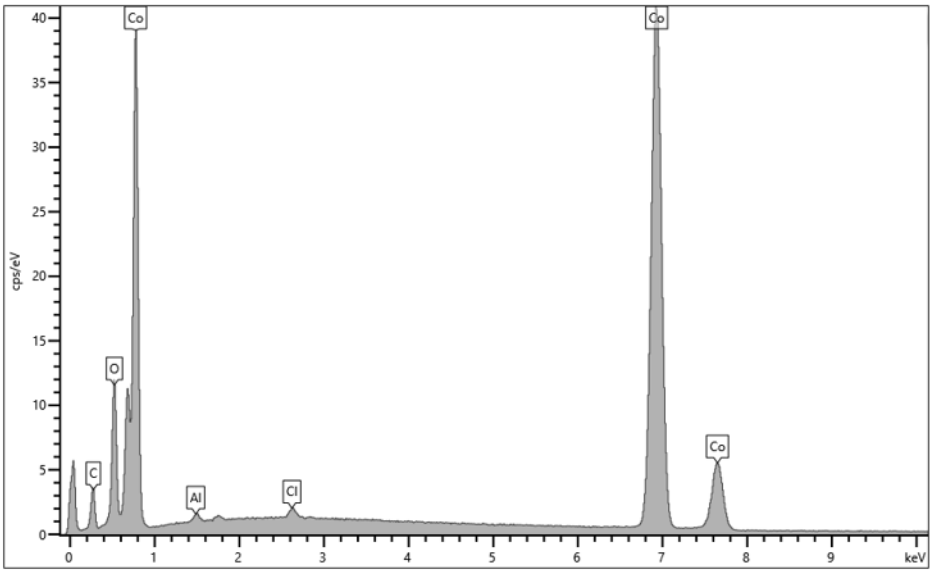

Figure S2. EDS spectrum of the Co-NPG_195 sample.

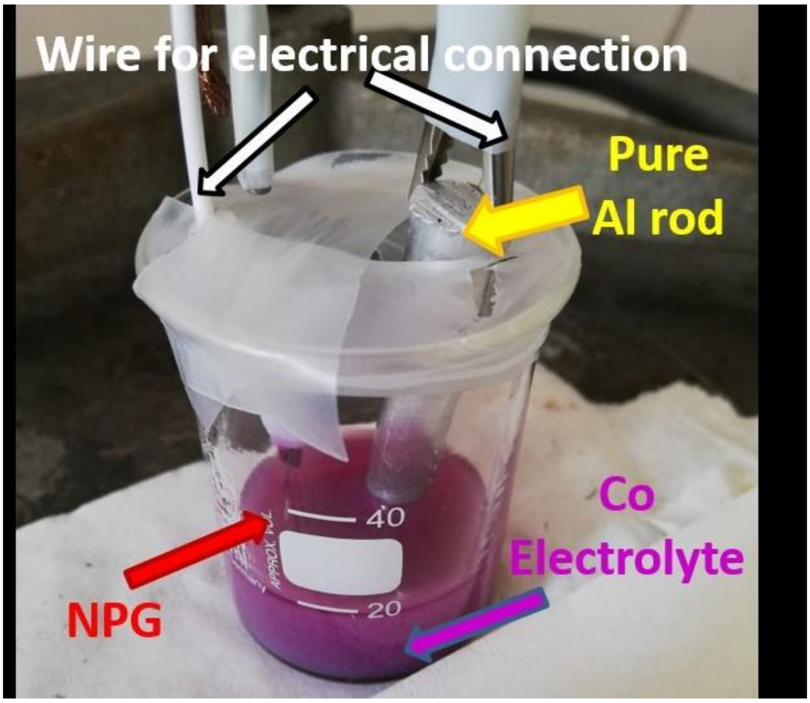

Figure S3. experimental setup for electroless Co deposition on NPG substrate.

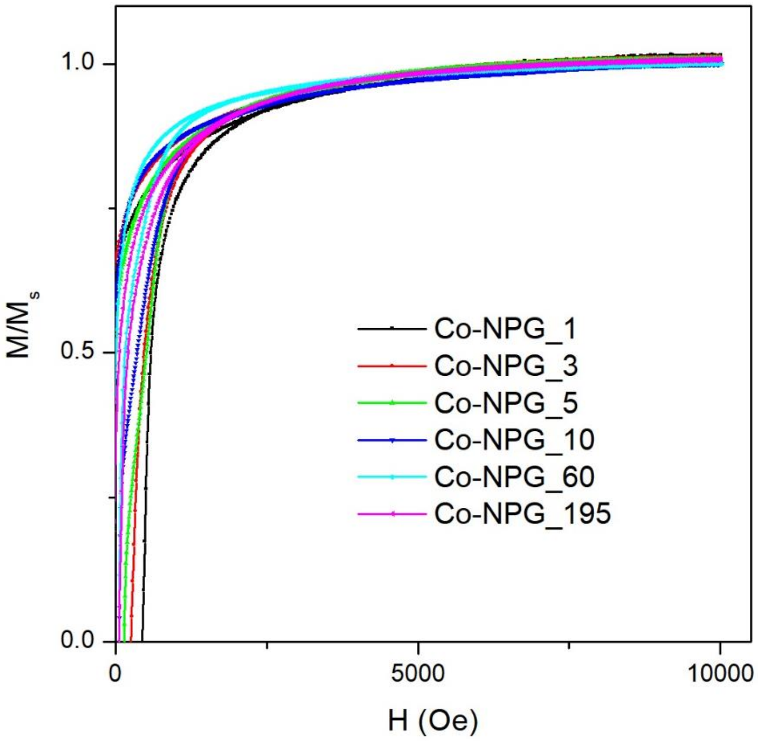

Figure S4. High-field magnification of the room temperature hysteresis loops for all hybrid Co/NPG heterostructures.
